# Are sex ratio distorting endosymbionts responsible for mating system variation among dance flies (Diptera: Empidinae)?

**DOI:** 10.1371/journal.pone.0178364

**Published:** 2017-06-13

**Authors:** Rosalind L. Murray, Elizabeth J. Herridge, Rob W. Ness, Luc F. Bussière

**Affiliations:** 1 Department of Biology, University of Toronto Mississauga, Mississauga, ON, Canada; 2 Ecology and Evolutionary Biology, University of Toronto, Toronto, ON, Canada; 3 Biological and Environmental Sciences, University of Stirling, Stirling, United Kingdom; University of Minnesota, UNITED STATES

## Abstract

Maternally inherited bacterial endosymbionts are common in many arthropod species. Some endosymbionts cause female-biased sex ratio distortion in their hosts that can result in profound changes to a host’s mating behaviour and reproductive biology. Dance flies (Diptera: Empidinae) are well known for their unusual reproductive biology, including species with female-specific ornamentation and female-biased lek-like swarming behaviour. The cause of the repeated evolution of female ornaments in these flies remains unknown, but is probably associated with female-biased sex ratios in individual species. In this study we assessed whether dance flies harbour sex ratio distorting endosymbionts that might have driven these mating system evolutionary changes. We measured the incidence and prevalence of infection by three endosymbionts that are known to cause female-biased sex ratios in other insect hosts (*Wolbachia*, *Rickettsia* and *Spiroplasma*) across 20 species of dance flies. We found evidence of widespread infection by all three symbionts and variation in sex-specific prevalence across the taxa sampled. However, there was no relationship between infection prevalence and adult sex ratio measures and no evidence that female ornaments are associated with high prevalences of sex-biased symbiont infections. We conclude that the current distribution of endosymbiont infections is unlikely to explain the diversity in mating systems among dance fly species.

## Introduction

Vertically transmitted symbiotic bacteria that are inherited from mother to offspring are common infections of arthropods [1–3]. These bacteria persist in host populations either as mutualists, by elevating aspects of host fitness, or as reproductive parasites, by manipulating host reproduction [2]. Whilst some reproductive parasites induce cytoplasmic incompatibility, which leaves host sex ratios unaltered, others are sex ratio distorters that increase the proportion of female offspring in the progeny of symbiont-infected females (reviewed in [2]). Although most sex ratio distorting symbionts of insects have modest impacts (e.g. [4,5]), some cause very strongly female-biased host population sex ratios (e.g. [6]).

A strong female bias in population sex ratio can profoundly alter the dynamics of sexual selection (e.g. [6–8]). When males become rare, male contests for mates can become less intense, and competition between females may also increase. Such changes in the relative numbers of sexually receptive individuals can affect the form and intensity of intrasexual competition, sexual conflict and mate choice, which in turn can affect the mating and parental roles within a species [9–13]. For example, in some butterfly species, symbiont-induced sex ratio distortion causes males to be scarce, resulting in female competition for access to mates [6], decreased per-mating male investment in ejaculates and nuptial gifts, and increased female polyandrous behaviour [14].

The dance flies (Diptera: Empididae: Empidinae) exhibit incredible interspecific mating system diversity [15]. Dance fly species vary substantially in courtship behaviour and sexual dimorphism. Unusually, the females of approximately one-third of dance fly species display several dramatic sex-specific ornaments, including pinnate leg scales, darkened or enlarged wings and inflatable abdominal air sacs [15]. Furthermore, most dance fly species form lek-like mating swarms in which males provide nuptial gifts to females (often prey items, but see [16]). In some taxa, the operational sex ratio (OSR) of these mating swarms is female-biased (e.g. [15,17–21]) such that females compete for access to male mates and the nuptial gifts they provide.

The ultimate evolutionary causes of these interspecific mating system differences across the dance flies remain largely unknown. One possibility is that reproductive parasites have caused female-biased population primary sex ratios in some species. Such biases could conceivably lead to competition among females for access to males, which could in turn favour the evolution of elaborate female sexual ornaments. Such symbiont-induced skewed sex ratios may have altered mating behaviour (see [6]), triggering the evolution of exaggerated morphological display traits in females of affected species. In this study we test for a link between the presence of putative sex ratio distorting symbiotic bacteria and the extreme mating system diversification within the Empidinae subfamily of dance flies (Diptera: Empididae).

Symbionts that influence arthropod host sex ratios belong to a diverse range of bacterial taxa [1]. One symbiont taxon that specializes in manipulating the reproductive biology of a wide diversity of arthropod hosts is *Wolbachia* [22–24]. *Wolbachia* infections are widespread, affecting up to two thirds of insect species [3,25]. Other symbiont taxa that contain sex ratio distorters include *Rickettsia* and *Spiroplasma* bacteria [1,2], which infect diverse arthropod hosts including spiders [26], ladybirds [5], Orbatid mites [27], *Drosophila* [28,29] and the Diptera superfamilies Muscoidea [30] and Empidoidea [31]. The prevalence of sex ratio distorting symbionts is highly variable; occasionally only a small proportion of a host population is infected [5], making them difficult to detect if small sample sizes are screened and suggesting that their incidence has been underestimated by many studies [5].

Forcing infected hosts to produce female-biased offspring sex ratios is adaptive for reproductive parasites because females alone transmit these maternally inherited symbionts to the next generation [2]. There are several different sex ratio distortion mechanisms, including male killing [32], feminization [33], and parthenogenesis induction [34]. Whilst there are many bacterial symbionts of arthropods that do not interfere with reproduction, sex ratio distorters exhibit a characteristic signature in host populations: they have higher prevalence in females than in males, because males originate mostly or wholly from uninfected females [2,3]. Further, if the infection prevalence of a sex ratio distorter is high, a female bias will be detectable in the adult population sex ratio (ASR) [2]. Such primary sex ratio biases might be responsible for interspecific diversity in the intensity of female contests for access to mates if the shifts in ASR are consistent predictors of shifts in the OSR. In particular, if sex ratio distorting symbionts are an important factor in the interspecific diversity in mating systems among dance flies, then those species with strong female contests (indicated by female biased OSRs) should feature strong and female biased infections.

In this study we assess the incidence of symbiont infection across 20 dance fly species. We investigate which infections may be sex ratio distorters by testing for female-biases in symbiont prevalence and by testing for population sex ratio biases using two independent collection methods. Then we test whether the presence of sex ratio distorters in dance fly species correlates with the evolution of costly sexually-selected display traits in females. We focus on the subfamily Empidinae (Diptera: Empididae), which contains three genera (*Hilara*, *Rhamphomyia* and *Empis*) with substantial mating system variation [21,35,36].

## Methods

### Sample collection and morphology measures

We collected 1515 dance fly specimens from vegetation using sweep nets from April to August of both 2011 and 2012 from the four locations (see Results for details). None of the species collected is protected or endangered and all samples were collected from public land that did not require specific permits for specimen removal. These specimens were our primary sources for measuring endosymbiont prevalence, while specimens from nearby mating swarms provided information on the (OSR) and were used to supplement sample sizes for determining symbiont prevalences where necessary (see below). Sample sizes varied based on species abundance at the time and location of sampling. We aimed to collect at least 20 females per species, which would provide a 90% chance of detecting symbiont infection rates of 12% prevalence or higher.

Ideally, to test for the presence of an individual sex ratio distorting bacterial infection, the primary sex ratio produced by infected females would be assessed. However, it is not known how or where dance flies lay their eggs, which makes measuring the primary sex ratio for these species particularly difficult. Instead, to estimate population-level sex ratio bias we measured the adult sex ratio (ASR) from specimens collected using two methods (Malaise traps and vegetation sweeps). We used Malaise traps to passively collect individuals flying through the habitat, which is an efficient and effective technique for collecting in multiple locations. Malaise trap samples were collected every three days during the collection period and stored in 70% ethanol.

Passive collection of specimens by Malaise traps is sensitive to any sex differences in movement (e.g., if males range more widely because of their hunting behaviours, or females range more widely because of their search for suitable oviposition substrates). To complement Malaise trap sampling, we also collected flies directly from flowers where both sexes congregate for nectivorous feeding. While both sexes can be observed on flowers, this method could also be biased if the sexes differ in rates of nectivory, (e.g., if one sex requires more fuel for long periods of swarming activity). Sweep net samples were collected daily throughout the season and across the local habitat and stored at -20°C.

When testing for the presence of endosymbionts, we used vegetation sweep net samples for which flies were stored individually and avoided using specimens stored in alcohol (i.e. Malaise trap samples) to prevent potential contamination from hosts being stored together. If we did not collect enough vegetation sweep net samples (fewer than 20 of each sex) for a particular species, we supplemented wherever possible with samples from species-specific mating swarms. Here, individuals collected from the mating swarms did not contribute to estimates of the ASR because individuals from the mating swarm represent a species OSR measure rather than population-level ASR measures, and we would expect these two sex ratio measures to differ [9]. We report all sex ratios as the proportion of males in a population.

Empidinae dance flies from many species are known to display one or more forms of sexually selected female-specific ornamentation, which can include darkened or enlarged wings, pinnate leg scales and inflatable abdominal sacs [15]. Within each type of ornament, there is considerable a variation in ornament expression: pinnate leg scales that differ in position within and among legs [37], wings that vary in size and colour patterns [19,38]; in some species inflatable abdominal sacs extend laterally as tubules [15,37] whilst in others they form lobes along the entire length of the abdomen [21]. With so much variation within ornament types, it seems unlikely that there was a single evolutionary origin for each of these traits. For the purposes of this study, we classified species as ornamented if females displayed at least one of these traits, and unornamented if females had none of these traits; we also categorized species into the type of ornaments that they displayed.

### Testing for endosymbiont prevalence

We extracted DNA using DNeasy animal tissue extraction kits (Qiagen, Valencia, CA) according to the manufacturer’s instructions. Whole samples were individually crushed using a mortar and pestle and then a small sample of each fly was removed for extraction while the remainder of the specimen was stored at -20°C. For each extraction, we pooled fly tissue in groups of five individuals of the same sex and species. The mitochondrial cytochrome oxidase subunit I (*COI*) region of each sample was amplified to confirm successful DNA extraction using primers LCO1490 and HCO2198 [39]. We used PCR to test for the presence of three symbionts, *Wolbachia*, *Rickettsia* and *Spiroplasma*, using the primers wsp81f –wsp691r [40], R1—R2 [41], and 27F –MGSO [42] respectively. PCR amplifications were carried out in 20μl reactions with 6.3μl ddH_2_O, 4μl 5X Taq Polymerase Buffer, 2.0μl of 25 mM MgCl_2_, 0.5μl of each primer (10μM), 1μl of 10 mM dNTPs, 1 unit of GoTaq DNA polymerase (Promega Cat. No. M830b), and 2–4μl of template DNA. We used a standard PCR protocol (2 min denaturation at 95°C followed by 35 cycles of 95°C for 60s, 54°C for 30s, 72°C for 30s and a final elongation at 72°C for 5m). We then visualized PCR amplicons on 1% agarose gels to score the presence or absence of a band. For each PCR reaction we ran a positive control (with a known sample infected with the symbiont being tested: a *Drosophila melanogaster* host for *Wolbachia*, and a successfully amplified dance fly host from our samples for *Spiroplasma* and *Rickettsia*) and a negative control (water). Where PCR amplification indicated a symbiont infection, we extracted DNA from each of the five individuals that made up the positive pooled sample and PCR amplified the individual specimens to get a precise measure of symbiont infection status for each individual fly.

### Statistical analyses

All statistical analyses were conducted using R v. 3.2.5 [43]. In order to test whether endosymbionts were affecting the host sex ratios, we estimated the ASR using both vegetation sweep netting and Malaise trap samples. We performed Pearson chi-square analyses to determine whether the sampling methods differed in the number of species and total specimens that were caught. Next, we tested for sex ratio bias (difference from 1:1) in each species using a binomial goodness of fit test for both Malaise and vegetation sweep netting separately.

To test for a relationship between symbiont presence and ASR, we fit generalized linear models with ASR coded as a two vector response (no. males and no. females collected with each sampling method), symbiont prevalence in each of the three symbionts tested (*Rickettsia*, *Spiroplasma* and *Wolbachia*) as a fixed effect and a quasibinomial family with a logit link function to limit the output in the interval between zero and one. We fit two models total, one each for the different collection methods.

Finally, to test whether symbiont infection prevalence bias predicted female-specific ornamentation across dance fly species, we fit two slightly different binomial generalized linear mixed effects models using the lme4 package [44]. The models differ in the measure of infection prevalence and sex-bias that were used to predict female ornamentation. We used female ornamentation as the response variable in these models. In order to check that a single ornament was not being influenced by symbiont prevalence, we coded each ornament (pinnate leg scales, abdominal inflation and wing dimorphism) separately, and compared it to a model with all ornaments grouped together. The models coding each ornament separately did not differ qualitatively from models with grouped ornaments. For simplicity, we report models with a binomial index of female ornamentation (present or absent) as our response variable below. For our first model we fit the total prevalence of infection across a host species, type of infection (endosymbiont taxa), and presence of female-biased infections (1 or 0) as fixed effects. For our second model, the difference in prevalence of infection between the sexes (female-male) and type of infection (*Rickettsia*, *Spiroplasma* or *Wolbachia*) were included as fixed effects. For both models, host species was fit as a random effect because the prevalence of each type of symbiont was measured in each host species such that a host species could be in the data set up to three times if it was infected with all three symbionts. We simplified the model by removing fixed effect terms and comparing the model deviance using a chi-square test.

## Results

### Dance fly sex ratio measures

The number of species collected, the sample size per species, and the direction of sex ratio bias differed depending on the sampling method (Table 1). Vegetation sweep counts ranged from 1 to 693 individuals per species and Malaise trap counts ranged from 2 to 1896 individuals per species. The different collection methods differed significantly in the number of species collected and the total number of specimens collected (Pearson chi-square, p<0.001 for both; Table 1). For ASR estimates from sweeping vegetation, most species did not differ from a 1:1 sex ratio except for *E*. *tessellata* and *R*. *longipes*, which both had female-biased sex ratios. Similarly for Malaise trap samples, most species did not differ from 1:1, but we found male-biased sex ratios for *E*. *nigripes* and *R*. *longipes*, and female-biased sex ratios for *R*. *dentipes* and *R*. *tibiella* (Table 1).

**Table 1:** Sex ratio estimates for 20 dance fly species from three genera: *Empis*, *Hilara and Rhamphomyia*. Adult sex ratios (ASR) calculated from two different sampling techniques and operational sex ratio estimates are displayed. Sex ratios shown as the proportion of males (larger values are more male-biased) followed by the lower and upper binomial confidence intervals (confidence level = 0.95). Deviations from 1:1 were calculated using an exact binomial goodness of fit test.

Species	Location	Vegetation ASR	Vegetation specimens (N)	Malaise ASR	Malaise specimens (N)	OSR^5^
***E*. *aestiva***	SCENE, UK^1^	0.54 (0.41, 0.67)	55	0.48 (0.42, 0.53)	365	0.34 (0.29, 0.39)
***E*. *borealis***	Aviemore, UK^2^	0.54 (0.37, 0.67)	41	NA	NA	0.44 (0.32, 0.56)
***E*. *grisea***	SCENE, UK	0.25 (0.04, 0.55)	8	0.54 (0.37, 0.68)	37	NA
***E*. *nigripes***	SCENE, UK	0.49 (0.44, 0.54)	397	0.64*** (0.59, 0.69)	339	0.46 (0.30, 0.62)
***E*. *stercorea***	SCENE, UK	0.35 (0.16, 0.55)	20	0.44 (0.31, 0.55)	64	NA
***E*. *tessellata***	SCENE, UK	0.23* (0.06, 0.47)	13	0.60 (0.47, 0.70)	70	0.71 (0.61, 0.81)
***H*. *chorica***	SCENE, UK	NA	1	NA	2	0.54 (0.52, 0.56)
***H*. *interstincta***	SCENE, UK	NA	1	NA	0	0.82 (0.63, 0.93)
***H*. *litorea***	Edinburgh, UK^3^	NA	0	NA	0	0.64 (0.62, 0.66)
***H*. *maura***	SCENE, UK	NA	2	NA	0	0.62 (0.45, 0.79)
***R*. *albohirta***	SCENE, UK	0.50 (0.15, 0.77)	8	0.59 (0.43, 0.73)	42	NA
***R*. *crassirostris***	SCENE, UK	0.83 (0.48, 0.96)	12	0.60 (0.48, 0.70)	75	0.34 (0.29, 0.39)
***R*. *dentipes***	SCENE, UK	0.73 (0.37, 0.90)	11	0.17** (0.05, 0.35)	23	NA
***R*. *longicauda***	Glen WIlliams, ON, Canada^4^	0.54 (0.40, 0.66)	56	NA	4	0.24 (0.20, 0.28)
***R*. *longipes***	SCENE, UK	0.40** (0.36, 0.44)	693	0.55*** (0.53, 0.57)	1896	0.71 (0.67, 0.75)
***R*. *nigripennis***	SCENE, UK	0.47 (0.37, 0.56)	121	0.70 (0.33, 0.89)	10	0.87 (0.54, 0.99)
***R*. *stigmosa***	SCENE, UK	0.48 (0.29, 0.65)	29	0.33 (0.08, 0.62)	9	0.57 (0.36, 0.78)
***R*. *sulcata***	SCENE, UK	NA	1	0.33 (0.05, 0.68)	6	0.63 (0.54, 0.72)
***R*. *tibiella***	SCENE, UK	0.63 (0.46, 0.76)	38	0.40* (0.32, 0.47)	160	0.59 (0.46, 0.74)
***R*. *umbripennis***	SCENE, UK	0.50 (0.15, 0.77)	8	0.50 (0.39, 0.59)	91	NA

SCENE: Scottish Centre for Ecology and the Natural Environment

GPS coordinates

^1^56.128557, -4.613103

^2^57.242442, -3.709810

^3^55.922339, -3.173880

^4^43.686497, 79.926098

^5^OSR values from [45]

*p<0.05

**p<0.01

***p<0.001

### Symbiont prevalence

**Fig 1:**
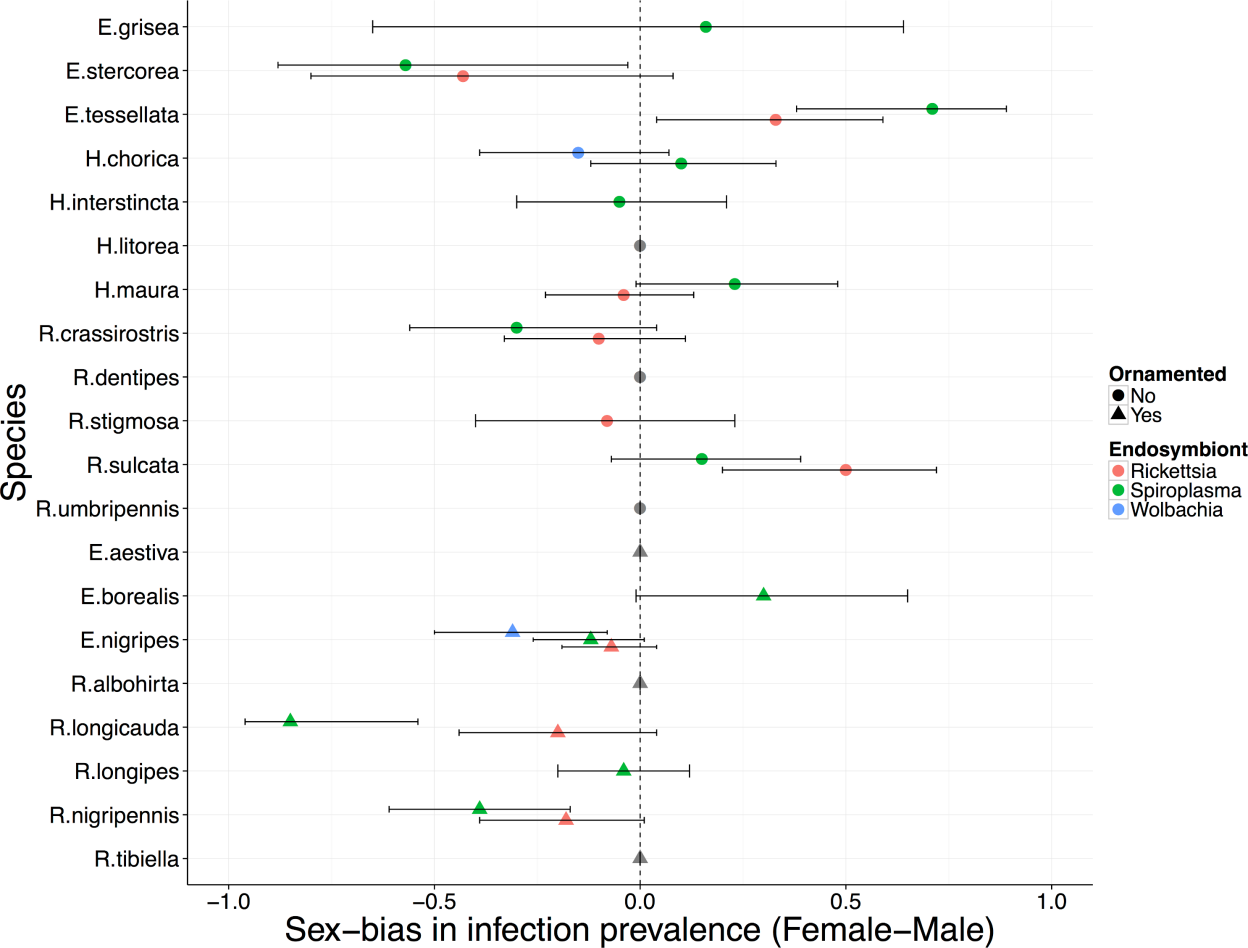
Forest plot displaying the sex-bias in endosymbiont prevalence across dance fly host species from three genera (*Empis*, *Hilara* and *Rhamphomyia*) with and without female-specific ornamentation. Circles indicate host species without female ornamentation, while triangles indicate species with ornaments. The endosymbiont taxa are identified by colour: red = *Rickettsia*, green = *Spiroplasma*, blue = *Wolbachia*. The error bars are the 95% confidence intervals around the sex-bias (difference in prevalence between males and females) calculated using the Wilson procedure with continuity correction.

We tested 719 individuals (using sweep net collections from vegetation and mating swarms) from 20 dance fly species for the presence of three commonly occurring and potentially sex ratio distorting symbionts, *Rickettsia*, *Spiroplasma* and *Wolbachia* (Table 2, Fig 1). Fourteen of the host species were infected with at least one symbiont. *Wolbachia* was found in two host species, *Rickettsia* in nine and *Spiroplasma* in thirteen (Table 2, Fig 1). We found eight species that were infected with two symbionts, and one, *E*. *nigripes*, where PCR indicated the presence of *Wolbachia*, *Rickettsia* and *Spiroplasma* bacteria. Most individuals harboured only a single symbiont; however, we found 17 hosts from six species were infected with two symbiont taxa, and two female *E*. *nigripes* hosts that were infected with all three symbionts we tested for (Table 2, Fig 1).

**Table 2:** Symbiont infections by *Wolbachia*, *Rickettsia* and *Spiroplasma* for 20 dance fly species from three genera (*Empis*, *Hilara* and *Rhamphomyia*) are shown along with the presence (1) or absence (0) of female-specific ornamentation displayed by each host species. Prevalence values shown are the proportion of each sex that was infected followed by the lower and upper binomial confidence intervals (confidence level = 0.95). Deviations from 1:1 were calculated using an exact binomial goodness of fit test. Coinfection is any individual host that was found to have more than one symbiont during screening.

Species	N	symbiont	prevalence in females	prevalence in males	coinfection^b^ (no. hosts infected, host sex, symbionts)	female ornament^c^
***E*. *aestiva***	56					1 (p)
***E*. *borealis***	20	*Spiroplasma*	0.00	0.30 (0.07,0.65)		1 (ws)
***E*. *grisea***	8	*Spiroplasma*	0.00	0.16 (0, 0.64)		0
***E*. *nigripes***	84	Wolbachia** Rickettsia *Spiroplasma*	0.71 (0.55,0.83) 0.07 (0.02,0.17) 0.12 (0.04,0.24)	0.40 (0.26,0.55) 0.00 0.00	2, f, wrs 3, f, ws 1, f, wr	1 (p)
***E*. *stercorea***	15	*Rickettsia* Spiroplasma*	0.43 (0.10,0.73) 0.57 (0.17,0.84)	0.00 0.00		0
***E*. *tessellata***	35	*Rickettsia* Spiroplasma***	0.00 0.00	0.33 (0.16,0.52) 0.71 (0.48,0.85)	4,m,rs	0
***H*. *chorica***	40	*Wolbachia* *Spiroplasma*	0.15 (0.04,0.33) 0.00	0.00 0.10 (0.02,0.27)		0
***H*. *interstincta***	40	*Spiroplasma*	0.15 (0.04,0.33)	0.10 (0.02,0.27)		0
***H*. *litorea***	40					0
***H*. *maura***	48	*Rickettsia* *Spiroplasma*	0.04 (0.01,0.18) 0.09 (0.02,0.23)	0.00 0.32 (0.16,0.52)		0
***R*. *albohirta***	8					1 (p, wc)
***R*. *crassirostris***	40	*Rickettsia* *Spiroplasma*	0.10 (0.02,0.27) 0.55 (0.31,0.73)	0.00 0.25 (0.09,0.45)	1, f, rs	0
***R*. *dentipes***	8					0
***R*. *longicauda***	40	*Rickettsia* Spiroplasma***	0.20 (0.06,0.39) 0.85 (0.60,0.95)	0.00 0.00	3, f, rs	1 (a, p)
***R*. *longipes***	56	*Spiroplasma*	0.04 (0.01,0.20)	0.00		1 (p)
***R*. *nigripennis***	54	*Rickettsia* Spiroplasma***	0.18 (0.07,0.35) 0.39 (0.16,0.52)	0.00 0.00	4, f, rs	1 (wc)
***R*. *stigmosa***	24	*Rickettsia*	0.08 (0.01,0.31)	0.00		0
***R*. *sulcata***	40	Rickettsia** Spiroplasma	0.00 0.00	0.50 (0.27,0.69) 0.15 (0.04,0.33)	1, m, rs	0
***R*. *tibiella***	33					1 (a, p)
***R*. *umbripennis***	30					0

*p<0.05

**p<0.01

***p<0.001 significant values indicate deviation from equitable prevalence for an endosymbiont in a given host taxa

^b^ f = female, m = male, w = *Wolbachia*, r = *Rickettsia*, s = *Spiroplasma*

^c^ for female-specific ornamentation, p = pinnate leg scales, a = inflatable abdominal sacs, wc = wing colour dimorphism, ws = wing size dimorphism

For those species that were infected, bacterial prevalence was variable, ranging from 0.08–56% for *Wolbachia*, 0.02–25% for *Rickettsia*, and 0.02–43% for *Spiroplasma*. Twenty of the 24 infections identified occurred in only one sex: seven only in males, and thirteen were found only in females (Table 2). Allowing a false discovery rate of 10% to account for multiple tests [46], out of the 14 infected host species, two had symbionts with significantly higher infection prevalence in males compared to females and four had a significantly higher infection prevalence in females compared to males (Table 2). These symbionts with female biased infection prevalence might be sex ratio distorting reproductive parasites: *E*. *nigripes* infected with *Wolbachia*, and *E*. *stercorea*, *R*. *longicauda* and *R*. *nigripennis* infected with *Spiroplasma* (Table 2).

### Sex ratios and infection status

We fit generalized linear models to test for an effect of the prevalence of a symbiont in a host population on the ASR calculated from two different sampling methods. We found that host species with increased prevalence of any of the three symbionts tested were not more likely to exhibit sex ratio bias when ASR was calculated from vegetation sweeps or Malaise trap sampling (Table 3).

**Table 3:** Results from quasibinomial generalized linear models investigating the effect of individual endosymbiont prevalence on dance fly adult sex ratio (ASR). Models were fit separately for the two sampling methods used to estimate ASR: Vegetation sweep netting and Malaise traps. Both models fit ASR as a two-vectored response variable (no. males, no. females) and the prevalence of each symbiont as predictors.

**Vegetation**	**estimate**	**se**	**p**
intercept	-0.31	0.11	0.01
*Rickettsia*	-0.08	0.10	0.42
*Spiroplasma*	3.36	0.03	0.23
*Wolbachia*	0.01	0.004	0.17
**Malaise**	**estimate**	**se**	**p**
intercept	0.06	0.07	0.43
*Rickettsia*	-0.08	0.08	0.34
*Spiroplasma*	0.05	0.03	0.15
*Wolbachia*	0.01	0.01	0.07

None of the four host species with female biased infections identified using a Fisher’s exact test had a corresponding female biased ASR estimated using either sampling method (Table 1). *Rhamphomyia longipes* and *R*. *tibiella* were the only host species for which we found a female biased ASR estimated from a large sample size (N>100; Table 1) for either collection method (note that *R*. *longipes* had a male-biased ASR using the other sampling method). However, these species did not exhibit a female biased symbiont infection prevalence, providing no evidence that that sex ratio distorting symbionts are responsible for these biases (*R*. *tibiella* harboured no symbionts, and while *R*. *longipes* had a very low prevalence female-only *Spiroplasma* infection (0.04; Table 2, Fig 1), the sex bias in the prevalence of this infection was not statistically significant).

### Symbionts and female-specific ornaments

To investigate the effect of symbiont prevalence on the evolution of female-specific ornaments we fit two binomial generalized linear mixed effects models with presence of female-specific ornaments as the response. If there is a relationship between symbiont prevalence and female-specific ornaments, we expected a significant interaction between prevalence of infection and/or infection sex-bias. More specifically, we would expect that high prevalence symbiont infections that are found more often in female hosts (female biased infections) should be the most likely predictors of female-specific ornaments. This is because female-bias in the host ASR caused by a high-prevalence sex ratio distorting symbiont could lead to the evolution of female ornaments. Our simplified models showed qualitatively similar results; there was no evidence that symbiont prevalence predicts the evolution of female-specific ornaments regardless of the way that infection prevalence was used in the model (Table 4).

**Table 4:** Results from binomial generalized linear mixed models investigating the effect of endosymbiont prevalence on female-specific ornament evolution. Two models were fit. One model tested for an effect of individual symbiont prevalence within host species and female-bias in infection on the evolution of female-specific ornaments with host species fit as a random effect (variance component: 2883). A second model fit sex-bias in infection prevalence (difference between female and male) within dance fly host species as a predictor with species fit as a random effect (variance component: 536.1).

**Individual symbiont prevalence**	**estimate**	**se**	**z**	**p**
intercept	-13.73	8.81	-1.56	0.10
prevalence	-2.63	20.90	-0.13	0.92
female-bias	3.36	16.86	0.20	0.84
**Sex-biased** **symbiont prevalence**	**estimate**	**se**	**z**	**p**
intercept	-9.77	4.47	-2.19	0.03
sex-bias prevalence	11.68	17.06	0.68	0.49

## Discussion

Dance flies from the subfamily Empidinae show remarkable variation in reproductive behaviour, with repeated evolution of exaggerated female-specific ornamentation [15]. While the focus of previous work in this group has been on nuptial gifts, and their potential role in increasing sexual selection on females, reproductive parasites present an important, and as yet untested, explanation for the prevalence of unusually high levels of competition between females in this group. Here we reported the incidence and prevalence of three bacterial symbiont taxa across 20 dance fly host species and tested whether the symbionts detected might play a role in distorting host sex ratio and thereby promoting the evolution of female-specific ornaments in the Empidinae.

Our screens for bacterial symbionts showed that there are many dance fly species that are infected with *Wolbachia*, *Spiroplasma* or *Rickettsia* (Fig 1, Table 2). We assessed two main pieces of evidence that each infection might be a sex ratio distorter: the symbiont should predominantly (or solely), infect females, and the host species should have a female biased population sex ratio. However we found little evidence that any of the symbionts we detected cause sex ratio distortion in their hosts. This is in contrast to other studies that found a high prevalence of sex ratio distorters in ladybird beetles (an insect group that is particularly predisposed to infection by reproductive parasites) using similar methods [5,47].

While it is possible that some of the symbionts we found could be low prevalence sex ratio distorters that we have limited power to detect, it nevertheless seems unlikely that the symbionts described here provide the selection pressure necessary to drive the repeated and unusual ornament evolution in female dance flies (Tables 3 and 4). If sex ratio distorting symbionts caused differences in sexual competition among female dance flies that resulted in the evolution of female ornamentation, then those host species for which we see strongly female biased OSRs (e.g. *E*. *aestiva*, *R*. *longicauda*; Table 1) should have detectable symbionts. However, given that we do not find evidence for sex ratio distorting symbionts (no female biased infection prevalence or female biased ASR: Tables 1 and 2) in species with female biased OSRs, there must be something else causing skew in the OSR across these species (see [48] for example).

### Symbiont prevalence

Many of the symbionts we found only infected a small proportion of the population (less than 3% of individuals; Table 2). However, because low prevalence infections are unlikely to cause strong population sex ratio distortion, this is unlikely to bias our study. We also did not test for all known symbiont taxa (e.g. Flavobacteria species [49] and *Cardinium* [50]) and instead limited our study to a subset of the sex ratio distorting symbionts. It therefore remains possible that other sex ratio distorting symbionts may infect these dance fly species.

Interestingly, we found several infections that occurred in males but were completely absent in females, including two that showed significant male-biased infection prevalence: *Spiroplasma* in *E*. *tessellata* and *Rickettsia* in *R*. *sulcata* (Table 2). If the symbionts we detected are indeed maternally inherited, then they must be present in the females of their host species. Possibly these symbionts may infect the sexes differently, perhaps remaining at low titres in females but proliferating in males. Alternatively, it remains possible that some of the symbionts we detected could be dietary contaminants: present within insect prey items that our dance flies had consumed. The nuptial gifts that males give to females are frequently other Diptera [15]. Dietary differences between males and females could therefore generate some of the sex biases in infection that we observed.

### Dance flies and symbionts

A previous study [31] that screened for *Rickettsia*, *Spiroplasma* and *Wolbachia* taxa across host species from the Empidoidea superfamily (to which the Empidinae subfamily belongs) included two species that overlap with our sample. Martin et al. [31] found an individual from each of *H*. *interstincta* and *E*. *nigripes* to be infected with *Rickettsia*. In our study, we found *E*. *nigripes* infections involving all three symbiont taxa, while *H*. *interstincta* was only infected with *Spiroplasma* (Table 2). Differences between these studies in host infection status likely result from variation in symbiont identity or prevalence across different host populations (continental Europe compared to UK populations) or environmental conditions (see [51]). Further screens investigating spatial and temporal patterns in dance fly symbiont incidence and prevalence across populations would be necessary to determine the dynamics of this system.

Our screens of symbionts also revealed several individuals with coinfections by more than one symbiont (Table 2). Previous studies have shown coinfection by multiple species of endosymbiont within a single host (e.g. [5,30]). Whilst we do not know what phenotypic effect these individual or coinfections have on their host, it is possible that coinfections occur in separate host tissue types and have different and/or interacting impacts on their hosts. Further study into where endosymbiont coinfections occur within a host and how coinfections influence a host’s phenotype compared to single infections would be useful.

For all of our statistical tests across dance fly species we treated each species as an independent replicate. Some of the host species likely share a recent evolutionary history, which might challenge our assumption of phylogenetic independence. However, it is unlikely that phylogenetic non-independence confounds our analysis because female ornamentation (in dance flies) and infection by sex ratio distorting symbionts (more generally) are both traits that evolve rapidly relative to the timescale of speciation: congeneric dance flies typically display divergent ornamentation characters [15] and the phylogenies of sex ratio distorting symbionts generally show little congruence with those of their hosts (e.g. [52]).

Finally, we tested for an effect of symbiont presence in our dance fly host species ASR measures using two different collection methods to estimate the ASR. We found that there was no relationship between symbiont prevalence ASR estimates (Table 3). Below we further discuss the results or our ASR measures and relate these findings to symbiont prevalence measures and implications for dance fly evolution, generally.

### Dance fly adult sex ratio estimates

We estimated the ASR using two sampling methods and showed variation in the number of species and specimens collected, the sample size per species and the direction of sex ratio bias between the two sampling methods (Table 1). Variation in ASR estimates is likely caused by differences in the phenology, ecology and behaviour between species and sexes [15,37,53]. While the majority of our ASR estimates reveal a sex ratio that is not significantly different from 0.5, two of the sex ratios from sweep net collections were female biased (*R*. *longipes* and *E*. *tessellata*), and four of our Malaise trap ASR estimates revealed significant deviations from an even sex ratio (Table 1). It should also be noted that our study focused on sex ratio estimates from one population of each species, and there is likely to be variation in population ASR measures across the spatial landscape.

Behavioural differences between the sexes could affect the proportion of males and females we collected using each sampling method. Notably, *R*. *longipes* had the largest sample size for both collection methods and was the only species that revealed sex ratio bias from both measures; however, the bias occurred in opposite directions. Furthermore, we found a female bias trend in the ASR measured for species from sweep netting vegetation (suggesting that females may engage in more nectivorous feeding, which is presumably necessary for long periods of swarming flight), while our ASR estimate based on Malaise traps significantly supported a male-biased ASR across species (suggesting males engage in more movement that makes them susceptible to Malaise trapping, perhaps as a consequence of their hunting behaviour to acquire nuptial gifts [18]). For example, in *Drosophila melanogaster*, males infected with *Wolbachia* have higher mating rates compared to uninfected males [54]. For dance flies, nuptial gifts to be presented to females during copulations can limit male mating rate [16]. If symbiont-infected males are attempting to mate more frequently, we would expect pressure on males to be more efficient at obtaining nuptial gifts. Within the dance flies, there are many species that use non-consumptive items as nuptial gifts and often this behaviour is considered ‘cheating’ (see [16] for discussion). If there is an increased mating rate in infected dance fly males, it would be interesting to measure the association between infection status and use of cheating (non-consumptive) nuptial gifts.

Despite finding little evidence to support the idea that reproductive parasites might be driving host ornament evolution (Table 4), we nonetheless have found several novel relationships between symbionts and dance fly hosts. There are many hypotheses to explain the widespread occurrence of symbionts including mutualism, cytoplasmic incompatibility or historic sex ratio distortion. We cannot exclude the possibility that other sex ratio distorters may have infected these species in the past, altering the pattern of sexual selection, or that the sex ratio phenotype of these symbionts could have changed (e.g. because of host resistance) since ornament expression evolved. Importantly, while we found no widespread evidence to support symbionts as drivers of ornament evolution, we do find four species (*E*. *nigripes*, *E*. *stercorea*, *R*. *longicauda* and *R*. *nigripennis*) with potential sex ratio distorting symbionts, as suggested by their female biased prevalence. However, only two of these species (*R*. *longicauda and E*. *nigripes*) display female-specific ornaments so it remains unlikely that symbionts are driving sex ratio distortion to the point that ornaments are evolving.

For those symbionts that are not candidates to be sex-ratio distorters, other factors must be maintaining them in their host populations. Symbionts may persist as parasites or beneficial mutualists within their hosts. While reproductive manipulation by inducing cytoplasmic incompatibility is a possible mechanism for these symbionts’ persistence within dance flies, it is also possible that symbionts act to protect their host against parasites (e.g. [55,56]) or provide resources (e.g. [57]).

### Conclusions

We tested for evidence that sex ratio distorting reproductive parasites have driven female-specific ornament evolution in the Empidinae. We found no evidence that reproductive parasites with strong impacts on host population sex ratio are common in this insect group. Furthermore, there was no association between the presence of putative sex ratio distorting endosymbionts and the presence of exaggerated female ornamentation. Further study with a focus on methods that allow for the primary or secondary sex ratio of the hosts to be measured would be helpful to more comprehensively measure sex ratio distortion in dance flies. In addition, research looking into spatial patterns of both the sex ratio of the dance fly hosts and the sex-specific prevalence of symbionts would be fruitful. While we cannot definitively rule out the possibility that historical infections by symbionts caused sex ratio distortion in ancestral populations, our data strongly suggest that female-biased OSRs and female-specific ornaments are being maintained in dance flies for some other reason than sex ratio distorting symbionts. Further research into sexual and natural selection within the mating swarms and reproductive behaviours of dance flies are important for fully understanding the diversity of reproductive phenotypes observed across this system.
